# Genetic analysis of Ghanaian G1P[8] and G9P[8] rotavirus A strains reveals the impact of P[8] VP4 gene polymorphism on P-genotyping

**DOI:** 10.1371/journal.pone.0218790

**Published:** 2019-06-26

**Authors:** Susan Afua Damanka, Chantal Ama Agbemabiese, Francis Ekow Dennis, Belinda Larteley Lartey, Theophilus Korku Adiku, Christabel Chika Enweronu-Laryea, George Enyimah Armah

**Affiliations:** 1 Department of Electron Microscopy and Histopathology, Noguchi Memorial Institute for Medical Research, College of Health Sciences, University of Ghana, Legon, Accra, Ghana; 2 School of Basic and Biomedical Sciences, University of Health and Allied Sciences, Ho, Ghana; 3 Department of Child Health, Korle Bu Teaching Hospital, College of Health Sciences, University of Ghana, Accra, Ghana; Universidad Nacional de la Plata, ARGENTINA

## Abstract

The World Health Organisation rotavirus surveillance networks have documented and shown eclectic geographic and temporal diversity in circulating G- and P- genotypes identified in children <5 years of age. To effectively monitor vaccine performance and effectiveness, robust molecular and phylogenetic techniques are essential to detect novel strain variants that might emerge due to vaccine pressure. This study inferred the phylogenetic history of the VP7 and VP4 genes of previously non-typeable strains and provided insight into the diversity of P[8] VP4 sequences which impacted the outcome of our routine VP4 genotyping method. Near-full-length VP7 gene and the VP8* fragment of the VP4 gene were obtained by Sanger sequencing and genotypes were determined using RotaC v2.0 web-based genotyping tool. The genotypes of the 57 rotavirus-positive samples with sufficient stool was determined. Forty-eight of the 57 (84.2%) had the P[8] specificity, of which 43 (89.6%) were characterized as P[8]a subtype and 5 (10.4%) as the rare OP354-like subtype. The VP7 gene of 27 samples were successfully sequenced and their G-genotypes confirmed as G1 (18/27) and G9 (9/27). Phylogenetic analysis of the P[8]a sequences placed them in subcluster IIIc within lineage III together with contemporary G1P[8], G3P[8], G8P[8], and G9P[8] strains detected globally from 2006–2016. The G1 VP7 sequences of the study strains formed a monophyletic cluster with African G1P[8] strains, previously detected in Ghana and Mali during the RotaTeq vaccine trial as well as Togo. The G9 VP7 sequences of the study strains formed a monophyletic cluster with contemporary African G9 sequences from neighbouring Burkina Faso within the major sub-cluster of lineage III. Mutations identified in the primer binding region of the VP8* sequence of the Ghanaian P[8]a strains may have resulted in the genotyping failure since the newly designed primer successfully genotyped the previously non-typeable P[8] strains. In summary, the G1, G9, and P[8]a sequences were highly similar to contemporary African strains at the lineage level. The study also resolved the methodological challenges of the standard genotyping techniques and highlighted the need for regular evaluation of the multiplex PCR-typing method especially in the post-vaccination era. The study further highlights the need for regions to start using sequencing data from local rotavirus strains to design and update genotyping primers.

## Introduction

Diarrhoea is one of the leading causes of childhood mortality and was responsible for nearly 500, 000 childhood deaths in 2015 [1]. *Rotavirus A* (RVA), a member of the genus *Rotavirus* and family Reoviridae [2], is the leading cause of viral diarrhoea in children under five years. In 2016, rotavirus was responsible for an estimated 128, 500 deaths of children under the age of five, of which 104, 733 deaths occurred in sub-Saharan Africa [3]. Prior to the introduction of the monovalent rotavirus vaccine—Rotarix into Ghana’s Expanded Programme on Immunisation (EPI) in May 2012, rotaviruses killed about 2,090 children below five years of age accounting for 3.6% of annual diarrhoeal disease deaths in Ghana (https://path.azureedge.net/media/documents/VAD_rotavirus_ghana_fs.pdf).

Rotaviruses are non-enveloped viruses which possess a triple-layered capsid surrounding a genome of 11 segments of double-stranded RNA (dsRNA). The genome encodes six structural viral proteins (VP1—VP4, VP6 and VP7) and six non-structural proteins (NSP1—NSP6) [2]. The outer layer is made up of two proteins, VP7 and VP4 against which neutralizing antibodies are produced. The VP6 forms the middle layer whereas the VP2 forms the core capsid that surrounds the genome of the virus. The VP3 of rotavirus caps the viral mRNAs and helps combat cellular innate antiviral defenses. The non-structural proteins play various functional roles during rotavirus replication [2]. A binary classification of RVA strains is based on the VP7 (glycoprotein, G-genotype) and VP4 (protease-sensitive protein, P-genotype) proteins [4, 5]. Currently, 36 G genotypes and 51 P genotypes have been reported in humans and animals (https://rega.kuleuven.be/cev/viralmetagenomics/virus-classification/rcwg). However, the majority of the global G and P genotypes of human rotaviruses detected are limited to G1P[8], G2P[4], G3P[8], G4P[8] and G9P[8] [6–8]. In addition, the G12 genotype, first detected in the Philippines in 1987 [9], emerged as a significant cause of rotavirus diarrhoea in children and have since persisted worldwide [10–19].

P[8] and P[4] RVAs have been identified as the first and second predominant P- genotypes, respectively, causing more than 90% of rotavirus-associated diarrhoea cases in many countries [20, 21]. Of note is the presence of P[8] in the world’s top six most frequently encountered RVA strains G1P[8], G3P[8], G4P[8], G9P[8], and G12P[8]. The P[8] genotype thus, accounts for 74% of the global prevalence of human VP4 rotavirus infections and hence its importance as an effective vaccine candidate [7]. It is therefore reasonable that the two widely used RVA vaccines, Rotarix a monovalent G1P[8] vaccine by GlaxoSmithKlein and RotaTeq a pentavalent vaccine containing the G1, G2, G3, G4, and P[8] genotypes by Merck, contain the P[8]-VP4 genotype as an antigenic component.

Phylogenetically, four distinct lineages of the P[8] genotype namely P[8]-Lineage I, P[8]-Lineage II, P[8]-Lineage III and P[8]-Lineage IV have been described [22, 23]. Current advances in molecular characterisation techniques have also distinguished the P[8] genotype into two antigenically distinct subtypes: P[8]a (P[8]-Lineage I–III) and P[8]b (OP354-like, i.e. P[8]-Lineage IV) [24]. Most of the world’s P[8] strains bear the P[8]a subtype; on the other hand, the P[8]b subtype is rare.

Widespread use of rotavirus vaccines could result in strain selection due to vaccine-induced selective pressure as demonstrated by Roczo-Farkas et al. [25] who noted changes in the diversity and distribution of circulating rotavirus genotypes in children less than 5 years who were hospitalized with rotavirus gastroenteritis after vaccine introduction in Australia. Since Ghana has introduced the Rotarix vaccine, the emergence of novel variants of circulating strains due to vaccine pressure may occur; therefore monitoring of changes in rotavirus genotype prevalence as well as identification of potential vaccine escape strains are important to improve vaccination strategies and the development of new rotavirus vaccines.

There is an abundance of molecular epidemiological data on the prevalence of both common and unusual G/P types determined by RT-PCR in Ghana. These studies described RVA strains with unusual G and P combinations and revealed the G9 genotype as an important emerging RVA in Ghana. In addition, it was also shown that G1P[8] RVA was replaced as the major circulating strain during the pre-vaccination era with G12P[8] as the most predominant strain post vaccine introduction. [26–29]. However, in-depth genetic comparisons at both the nucleotide and phylogenetic level has been rarely performed to detect if any the emergence of novel variants of the commonly detected strains in the country. Enweronu-Laryea et al. [30] employed a RT-PCR based genotyping method to characterize the G and P genotypes of RVA strains from stool samples collected from children with diarrhoea during the WHO sponsored surveillance study in Ghana. Their study recorded a substantial number of uncharacterized G (135/876) and P (167/876) genotypes. In this study, we determined the genotypes of previously non-typeable strains based on nucleotide sequence data and provided insight into the evolutionary history of the VP7 and VP4 genes by phylogenetic analysis. We also examined the effect of VP8* P[8] genotype diversity on the routinely used RT-PCR based genotyping techniques and resolved challenges associated with P[8]-genotyping failure.

## Materials and methods

### Ethical approval and background of study samples

The study protocol was approved by the Institutional Review Board, Noguchi Memorial Institute for Medical Research, Legon, Accra, Ghana. A total of 57 RVA positive samples analysed in this study were collected from children <5years who were hospitalized with a primary diagnosis of acute gastroenteritis between 2007 and 2011. The G-genotypes of these 57 samples were previously reported in studies carried out by Enweronu-Laryea et al. [30], Damanka et al. [31], [32] and Binka et al. [33]. The distribution of the G-genotypes were as follows: G1 (38), G2 (3), G6 (1), and G9 (15). Their P-types could however not be determined using the routine genotyping primers.

### RNA extraction, reverse transcription polymerase chain reaction and Sanger sequencing

Total RNA was extracted from 10% stool suspension by the phenol/chloroform method as described by Steele and Alexander [34] and the RNA purified with an RNaid kit (Bio101, Inc., California, USA) according to the manufacturer’s protocol. The RNA was reverse transcribed and amplified using the VP4 gene consensus primer set VP4F/VP4R [35]. The reaction was carried out with an initial reverse transcription step at 42°C for 30 minutes, followed by initial denaturation at 94°C for 2 minutes, 30 cycles of amplification (denaturation at 94°C for 1 minute, annealing at 42°C for 2 minutes and extension at 72°C for 3 minutes) and a final extension at 72°C for 7 minutes in a thermal cycler (Eppendorf, Hamburg, AG). The corresponding VP7 gene of the samples were amplified using the VP7-F/VP7-R consensus primers [35] and the same PCR cycle conditions.

The amplicons were purified with the QIAquick PCR purification kit (Qiagen/Westburg) according to the manufacturer’s protocol and sequenced in the forward and reverse directions using the respective amplification primers and the BigDye Terminator cycle sequencing kit v.3.1 (Applied Biosystems). The cycle sequenced products were purified, using ethanol-sodium acetate precipitation method, dried under vacuum and reconstituted in Hi-Di formamide. DNA sequences were determined using ABI 3130xl genetic analyser (Applied Biosystems, Foster City, CA).

### Sequence and phylogenetic analyses

Near-full-length VP7 and VP8* portion of the VP4 gene were obtained by combining the sequences obtained from the forward and reverse reactions. The genotypes were determined for the VP8* portion and confirmed for the G-types using the online RotaC v2.0 RVA genotyping tool [36]. Using the Basic Local Alignment Search Tool (BLAST) and the rotavirus resource in virus variation available at the NCBI website [37, 38], reference sequences for phylogenetic analysis were retrieved. Multiple sequence alignment was carried out on the datasets and the best fit nucleotide sequence substitution models were determined using MEGA version 6.06 [39].

Based on the best fit nucleotide substitution models with the lowest Bayesian Information Criterion scores [40], i.e. Tamura 3-parameter model with gamma distribution (T92+G) for the VP8* and G9 VP7 datasets and T92 + G + Invariant sites for the G1 VP7 dataset, maximum likelihood phylogenetic trees for the VP8* and the VP7 gene sequences were constructed using with 1000 bootstrap replicate trials. Lineages defined in the G9 VP7 phylogenetic tree were based on designations by Doan et al. [41] whereas those identified in the G1 VP7 and P[8] VP4 trees were based on designations by Cunliffe et al. [23] and Do et al. [42].

## Results

### G/P types determined based on sequence data

All 57 rotavirus-positive samples had enough bulk stool specimens for further analysis in this study. The VP8* fragment of the VP4 genes of these samples were successfully sequenced and genotypes were determined. Forty-eight of the 57 (84.2%) had the P[8] specificity, of which 43 (89.6%) were characterized as P[8]a subtype and 5 (10.4%) as the rare P[8]b (OP354-like) subtype. The P-genotypes of the remaining 9/57 samples were P[6]: 7(12.3%), P[4]: 1(1.75%), and P[14]: 1(1.75%). Out of the 43 P[8]a sequences, 39 (Table 1, S1 Table) were of appreciable length (nucleotide position 178–787 of the VP8* portion of the VP4 gene) and were included for further sequence comparison and phylogenetic analysis. Of these 39 successfully sequenced P[8]a samples, the corresponding VP7 gene of only 27 of them could be sequenced even though their G-genotypes were known through the routine PCR genotyping method. These G-genotypes were confirmed as G1 (18/27) and G9 (9/27) upon sequencing (Table 1, S1 Table).

**Table 1:** Genotypes and lineages determined for the VP7 and VP4 of previously non-typeable strains.

G/P type	Sample	Year of collection	VP7 gene Lineage	VP4 gene Lineage
**G1P[8]a**	GHA-00240/DC	2007	G1; sequence ND	P[8]-Lineage III
	GHA-00319/DC	2008	G1; sequence ND	P[8]-Lineage III
	GHA-00328/DC	2008	G1; sequence ND	P[8]-Lineage III
	GHA-00378/DC	2008	G1; sequence ND	P[8]-Lineage III
	GHA-00532/DC	2008	G1; sequence ND	P[8]-Lineage III
	GHA-00324/DC	2008	G1; sequence ND	P[8]-Lineage III
	GHA-00495/DC	2008	G1; sequence ND	P[8]-Lineage III
	GHA-00141/PML	2008	G1; sequence ND	P[8]-Lineage III
	GHA-00329/DC	2008	G1; sequence ND	P[8]-Lineage III
	GHA-5059/EB	2009	G1-Lineage I	P[8]-Lineage III
	GHA-00592/DC	2009	G1-Lineage I	P[8]-Lineage III
	GHA-00850/DC	2010	G1-Lineage I	P[8]-Lineage III
	GHA-0099/M	2010	G1-Lineage I	P[8]-Lineage III
	GHA-0123/P	2010	G1-Lineage I	P[8]-Lineage III
	GHA-0139/P	2010	G1-Lineage I	P[8]-Lineage III
	GHA-0028/K	2010	G1-Lineage I	P[8]-Lineage III
	GHA-0021/K	2010	G1-Lineage I	P[8]-Lineage III
	GHA-00892/DC	2010	G1-Lineage I	P[8]-Lineage III
	GHA-00840/DC	2010	G1; sequence ND	P[8]-Lineage III
	GHA-009I9/DC	2010	G1-Lineage I	P[8]-Lineage III
	GHA-474/AG	2010	G1-Lineage I	P[8]-Lineage III
	GHA-00845/DC	2010	G1-Lineage I	P[8]-Lineage III
	GHA-00702/PML	2010	G1-Lineage I	P[8]-Lineage III
	GHA-00759/PML	2010	G1-Lineage I	P[8]-Lineage III
	GHA-00800/PML	2010	G1-Lineage I	P[8]-Lineage III
	GHA-00789/PML	2010	G1-Lineage I	P[8]-Lineage III
	GHA-00796/PML	2010	G1-Lineage I	P[8]-Lineage III
	GHA-00793/PML	2010	G1-Lineage I	P[8]-Lineage III
	GHA-00713/PML	2010	G1; sequence ND	P[8]-Lineage III
	GHA-0093/M	2010	G9-Major sub-lineage III	P[8]-Lineage III
	GHA-00293/LA	2010	G9-Major sub-lineage III	P[8]-Lineage III
	GHA-00886/DC	2010	G9-Major sub-lineage III	P[8]-Lineage III
	GHA-00894/DC	2010	G9-Major sub-lineage III	P[8]-Lineage III
**G9P[8]**	GHA-0176/P	2010	G9-sequence ND	P[8]-Lineage III
	GHA-00710/PML	2010	G9-Major sub-lineage III	P[8]-Lineage III
	GHA-00802/PML	2010	G9-Major sub-lineage III	P[8]-Lineage III
	GHA-00801/PML	2010	G9-Major sub-lineage III	P[8]-Lineage III
	GHA-00810/PML	2010	G9-Major sub-lineage III	P[8]-Lineage III
	GHA-00784/PML	2010	G9-Major sub-lineage III	P[8]-Lineage III

ND: Sequence Not determined

### Sequence analyses and phylogenetic inference

#### Sequence analyses

The nucleotide and amino acid sequence diversity of the VP8* fragment of the VP4 gene among the Ghanaian P[8]a strains was relatively high even between strains detected from the same place and year, ranging from 0% - 5% and 0% - 9.4%, respectively. Specifically, strains from DC/2008 varied from 0%– 2.9%, those from DC/2010 varied from 0.2% - 3.2%, those from PML/2010 varied from 0.7% - 3.9%, those from EB/M/P/2010 varied from 0% - 2.9% (S2 and S3 Tables). Similarly, the G9 VP7 genes were 0% - 3.6% and 0% - 3.8% diverse at the nucleotide and amino acid levels respectively. On the other hand, the G1 VP7 genes were relatively closely related with nucleotide and amino acid diversity ranging from 0% - 1.8% and 0% - 2.9% respectively.

#### Phylogenetic inference of the P[8] VP8* sequences

**Fig 1:**
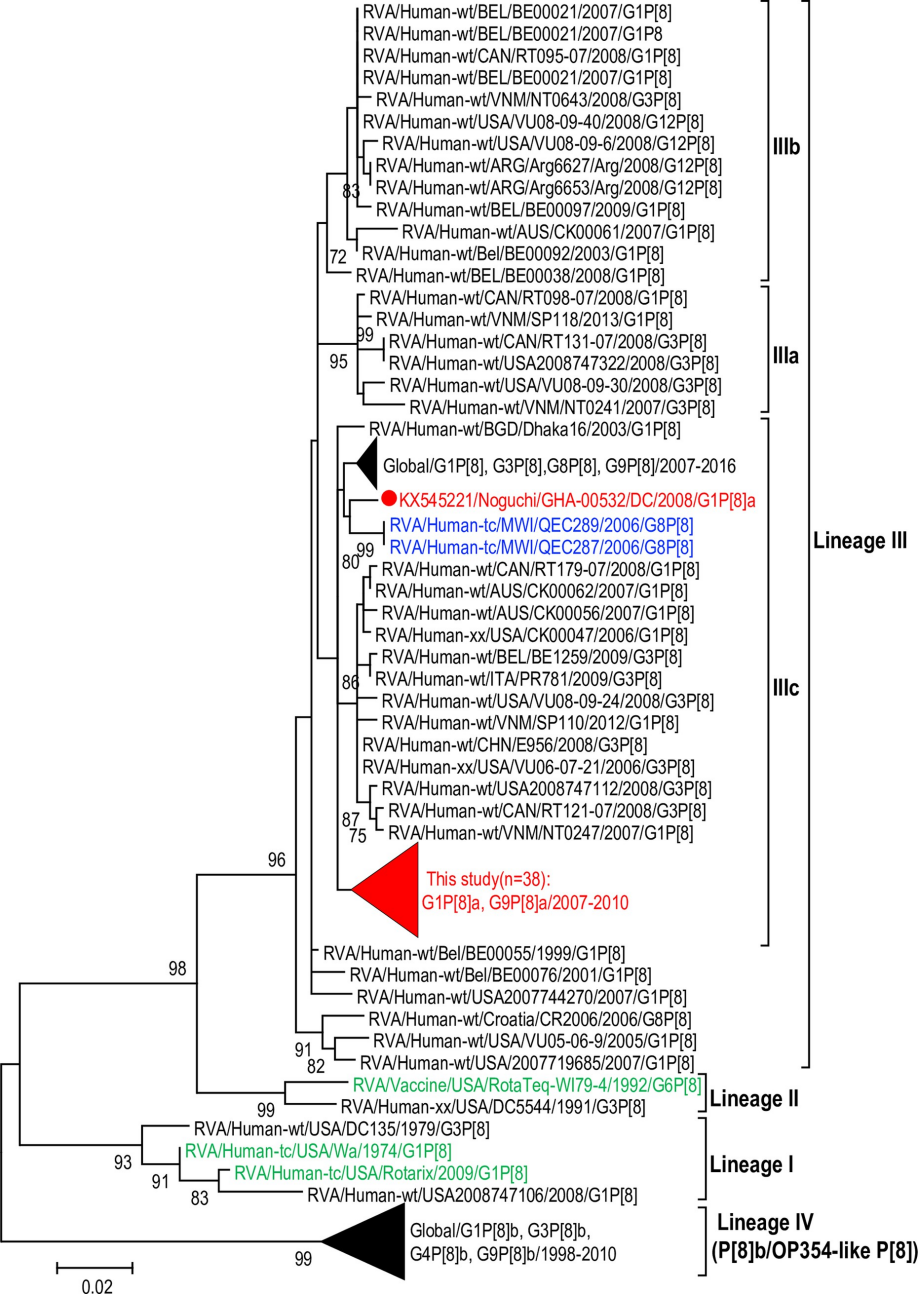
Phylogenetic tree of the VP8* fragment of the VP4 genes of Ghanaian P[8]a strains and published P[8]a strains constructed using maximum likelihood method with Mega 6.06 software. P[8]a strains analysed in the present study are indicated in red font. The prototype G1P[8] strain Wa and the P[8] vaccine strains in Rotarix and RotaTeq are in green font. Phylogenetic tree was supported statistically by bootstrapping with 1000 replicates. The scale bar at the bottom of the tree indicates genetic distance expressed as nucleotide substitutions per site. Percentage bootstrap support values ≥70% are shown.

Phylogenetic analysis of the VP8* fragment of the VP4 gene using sequences available in the DNA databases maintained the previously established four distinct lineages designated lineage I–lineage IV (Fig 1). The P[8]a sequences of the study strains belonged to sub-cluster IIIc within lineage III (Fig 1). This sub-cluster also included contemporary G1P[8], G3P[8], G8P[8], and G9P[8] strains detected from across the world from 2006–2016. Within sub-cluster IIIc, strain GHA-00532/DC/2008/G1P[8]a (indicated with a red dot) clustered separately from the rest of the Ghanaian P[8]a sequences but was closely related to the VP4 of two Malawian G8P[8] detected in 2006 (indicated in blue font). The phylogenetic analysis clearly showed that the study strains have a different evolutionary pathway from the P[8] sequences of historical strains such as the prototype G1P[8] strain Wa, and the vaccine strains of Rotarix and RotaTeq-WI79-4 (indicated in green font).

#### Phylogenetic inference of the G1 and G9 VP7 sequences

**Fig 2:**
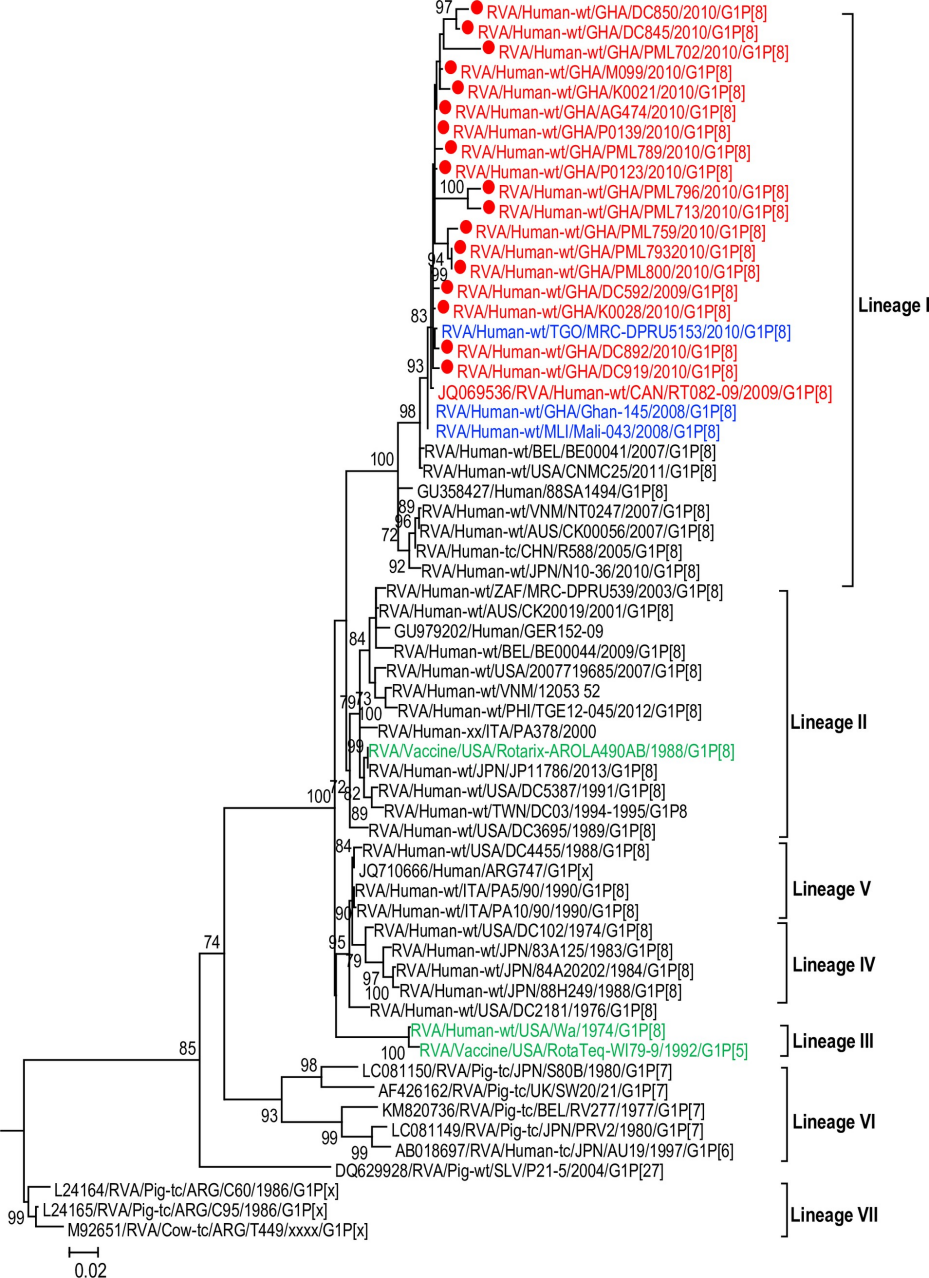
Phylogenetic tree of the VP7 sequences of G1P[8] strains and reference G1 strains retrieved from the GenBank database. The tree was constructed using maximum likelihood method with Mega 6.06 software. G1 strains analysed in the present study are indicated in red font. The prototype G1P[8] strain Wa and the P[8] vaccine strains in Rotarix and RotaTeq are in green font. The closest G1 sequences from African rotaviruses to our study strains are indicated in blue font. The phylogenetic tree was supported statistically by bootstrapping with 1000 replicates. The scale bar at the bottom of the tree indicates genetic distance expressed as nucleotide substitutions per site. Percentage bootstrap support values ≥70% are shown.

**Fig 3:**
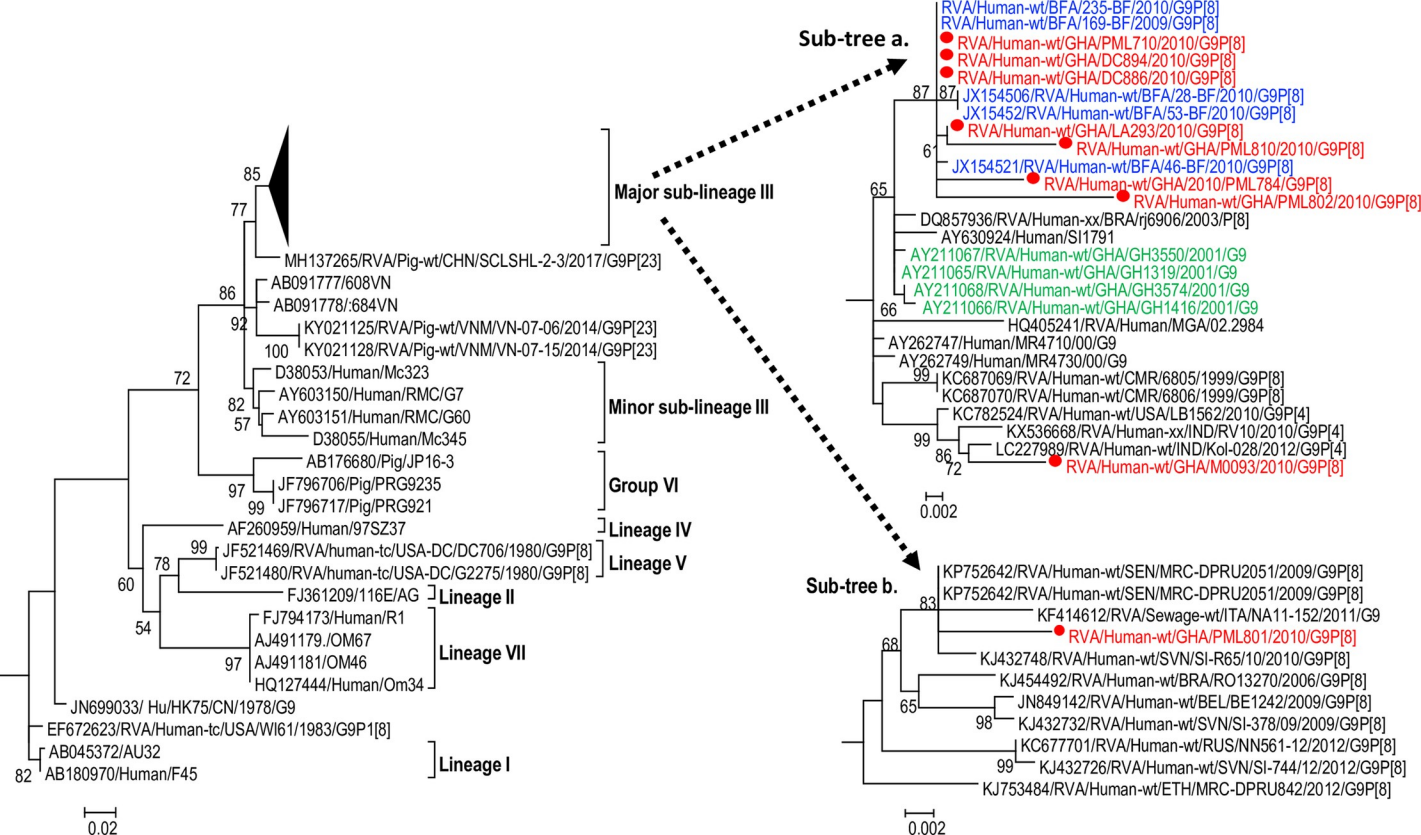
Phylogenetic tree of the VP7 sequences G9P[8] strains and reference G9 strains retrieved from the GenBank database. The tree was constructed using maximum likelihood method with Mega 6.06 software. G9 strains analysed in the present study are indicated in red font. The historical G9 strains reported from Ghana by Armah et al. [28] are indicated in green font. The closest G9 sequences from African rotaviruses to our study strains are indicated in blue font. The phylogenetic tree was supported statistically by bootstrapping with 1000 replicates. The scale bar at the bottom of the tree indicates genetic distance expressed as nucleotide substitutions per site. Percentage bootstrap support values ≥70% are shown.

Phylogenetic analysis of the G1 and G9 VP7 sequences of both human and porcine origin from the global rotavirus database resulted in previously established seven distinct lineages designated; lineage I–lineage VII (Figs 2 and 3). The G1 VP7 sequences of the study strains belonged to lineage I which is made of global G1 sequences of only contemporary human RVA strains detected from 2006–2016. Within lineage I, our study strains formed a monophyletic cluster with African G1P[8] strains detected previously in Ghana and Mali during Rotateq vaccine trial [43] and neighbouring Togo (indicated in blue font).

The G9 VP7 sequences of the study strains belonged to the major sub-cluster of lineage III which consists of global G9 sequences of both historical and contemporary human RVA strains. Within the major sub-lineage III, the study strains formed a monophyletic cluster (Fig 3 sub-tree a.) with African G9 sequences from neighbouring Burkina Faso (indicated in blue front) away from G9 sequences of Ghanaian G9 strains detected in 2001 (indicated in green front). It was noted that the VP7 sequence of the Ghanaian G9 study strain RVA/Human-wt/GHA/PML801/2010/G9P[8] had a different evolutionary history since it belonged to a sub-cluster consisting of strains from Africa, South America and Europe (Fig 3 sub-tree b).

#### Investigation of VP4 genotyping failure

**Fig 4:**
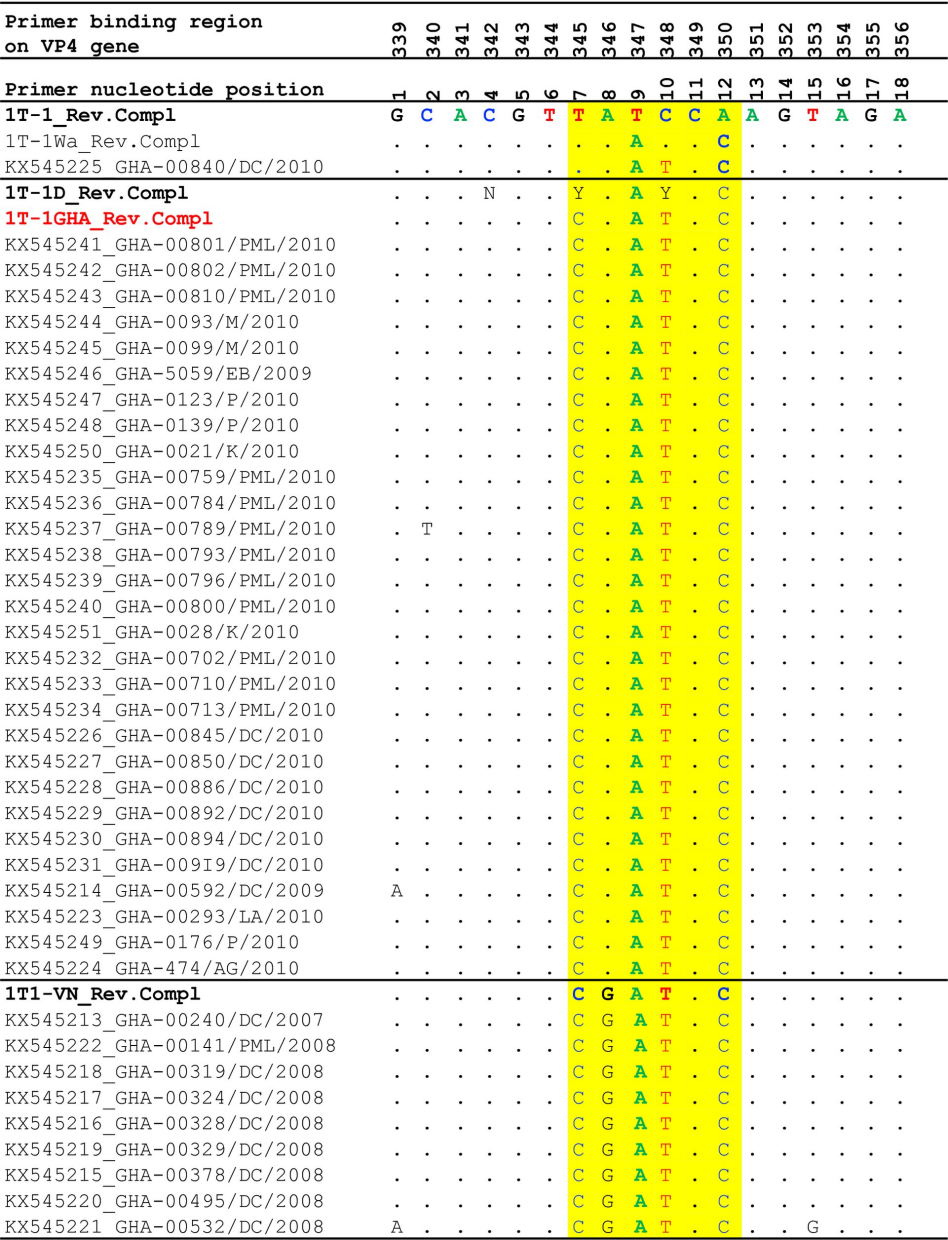
Alignment of VP8* fragments of the VP4 gene sequences of the Ghanaian P[8]a rotavirus strains and the reverse complementary sequences of the routinely used P[8] primers, 1T-1, 1T-Wa, 1T1-VN and the degenerate primer, 1T-1D. Dots indicate consensus with the primer and the yellow highlight indicates the region of variability of the primer binding site. Abbreviations: N: A or C or G or T; Y: C or T and R: A or G.

To investigate possible primer mismatches that led to the previously reported genotyping failure [30], 1T-1 as well as alternative P[8] genotyping primers namely 1T-1Wa, 1T1-VN, 1T-1D developed by WHO Rotavirus Collaborating Centers and Regional Laboratories were aligned with the P[8]a sequences. The following nucleotide mismatches were observed. First, strain GHA-00840 showed two mismatches when aligned with the routinely used P[8]-genotype specific primer 1T-1 at nt 10 (C→T) and 12 (A→C) and one mismatch at nt 10 (C→T) when aligned with the alternative primer 1T-1Wa primer (Fig 4).

Twenty-nine of the P[8]a sequences consistently showed three mismatches at nucleotides 7 (T→C), 10 (C→T) and 12 (A→C) (Fig 4) upon alignment with 1T-1. These twenty-nine samples also showed—two consistent mismatches at nucleotides; 7 (T→C),—and 10 (C→T) upon comparison with 1T-1Wa (Fig 4). Also, a mismatch at nt 8 (G→A) was identified when aligned with the 1T1-VN primer. However, an alignment with the 1T-1D degenerate primer (CAN GTY AAY CCA GTA GA) showed 100% complementarity with the Ghanaian P[8]a when the ambiguous codes N, Y, and Y were respectively substituted with C, C, and T (Fig 4). The new primer 1T-1GHA was ordered and tested by PCR against the non-typeable strains which were successfully genotyped as P[8].

Additionally, nine of the strains showed five consistent mismatches at nucleotide (nt) 7 (T→C), 8 (A→G), 9 (T→A), 10 (C→T) and 12 (A→C) when aligned with 1T-1 primer (Fig 4). These nine strains also showed three nucleotide mismatches at nt 7 (T→C), 8 (A→G) and 10 (C→T) when aligned with the 1T-1Wa primer (Fig 4) and 100% complementarity when aligned with the 1T1-VN primer except for strain GHA-00532/DC/2008 which had additional mismatches at position 1 (G→A) and 15 (T→G). Upon comparison with the degenerate primer 1T-1D (CAN GTY AAY CCA GTA GA), a single nucleotide mismatch at nt 8 (A→G) was detected.

## Discussion

Reverse transcription polymerase chain reaction genotyping methods used in rotavirus surveillance studies, have been useful in monitoring circulating strains and identifying rare and emerging strains worldwide. Advances in rotavirus molecular assays such as sequence analysis of rotavirus genes provide us the opportunity to i) genotype non-typeable rotavirus strains that have mutated hence might be recognized by the routine genotyping primers and ii) understand their evolutionary histories using molecular phylogenetic inferences. Our previous study on Ghanaian rotavirus strains revealed that a considerable proportion of the RVAs could not be G and/or P genotyped by the routine multiplex RT-PCR strategy by Gouvea et al. [4] and Gentsch et al., [5]. In this study, sequence data obtained by Sanger sequencing showed that the selected P-non-typeable strains possessed the G1P[8] and G9P[8] genotypes.

Circa 1999–2000, G1P[8] strains were rarely reported in Ghana while the G9P[8] strains emerged and predominated in the 4^th^ quarter of 1999 [28, 44]. From 2005 however, G1P[8] strains became the most prevalent strains accounting for 80% of strains from the Kasena Nankana district [45]. The G1 strains consistently remained prevalent while the G9 strains became rarely detected in Ghana [26, 46]. We inferred the phylogenetic history of the G1 and G9 VP7 sequences and noted that the strains characterized in this study were not very different from previously reported ones by Armah et al. (G9) [28] and Heylen et al. (G1P[8]) [43] from Ghana. There was evidence that our strains and those reported in the past diverged from a common ancestor. The little diversity observed can be considered typical taking into consideration the evolutionary rates of G1 VP7 gene (0.93 x10-^3^ nucleotide substitutions/site/year (Highest posterior density interval: 0.68 x10-^3^–1.18 x10-^3^) [47] and G9 VP7 gene (1.87 x10-^3^ nucleotide substitutions/site/year (Highest posterior density interval: 1.45 x10-^3^–2.27 x10-^3^) [11].

Phylogenetic analysis of P[8]—VP4 sequences from the global rotavirus database maintained that P[8] rotaviruses could be divided into the four previously established distinct phylogenetic lineages: P[8]-lineage I, P[8]-lineage II, P[8]-lineage III and P[8]-lineage IV. All the Ghanaian strains were grouped into lineage III and seemed to be related to African, Australian and European strains detected between 2006 and 2014, indicating that they shared common ancestral strains (Fig 1). In the phylogenetic tree, the Ghanaian strains clustered in P[8]-lineage III which differed from the lineages of vaccine strains (Rotarix and RotaTeq) which clustered in P[8]-lineage I and II respectively). This indicated that the evolutionary relationship between the P[8]-lineage of human RVAs detected in Ghana and the two vaccine strains are different.

Sequence comparison of the VP4 genes revealed mismatches at the P[8] primer (1T-1) binding site which we considered to be responsible for the genotyping failure. The substantial numbers of incompletely genotyped strains could be attributed to genetic drift due to the accumulation of point mutations or the lack of specific oligonucleotide primers for amplifying novel strains [48]. Majority of genotype specific primers used in multiplex PCR were designed from the sequences of strains detected in the 1980s and may have sequence mismatches in the primer binding region of the target gene of more recent rotavirus strains. Series of mutations were detected at the primer binding sites of the P[8] templates (our strains) thus decreased the primer binding probability, which explains the initial failure of the primers to bind to the P[8]a sites. Iturriza-Gomara and colleagues previously reported mismatches in the primer binding site of P[8] strains that resulted in genotyping failure [48]. Sequence based genotyping has helped determine the genotypes of more than 70% of non-typeable strains detected during the surveillance period thus reducing the percentage of non-typeable strains. It is interesting to note that majority of the VP4 strains characterized in this study belonged to the P[8]a subtype, an essential component of both Rotarix and RotaTeq vaccines. Failure to determine the P[8]a subtypes in our previous study indicated extreme simplification of the previous RT-PCR based genotyping system employed in our Regional Reference laboratory. The successful determination of the P[8]a genotype by sequencing showed that the prevalence of these strains were underestimated during the WHO sponsored rotavirus surveillance study in Ghana. A study in the United Kingdom showed that a degenerate version (1T-1D) of the P[8]-specific primer (1T-1) allowed strains previously non-typeable due to point mutations at the primer binding site to be P- typed by RT-PCR [33]. This study met with methodological challenges such as inter-primer suppression when employing a standard multiplex RT–PCR procedure using the commonly used P[8]-specific primer (1T-1) [20] and VP4 genotyping strategy developed by WHO Rotavirus Collaborating Centers and Regional Laboratories using 1T-1Wa, 1T1-VN (http://apps.who.int/iris/bitstream/handle/10665/70122/WHO_IVB_08.17_eng.pdf;jsessionid=8C5703F323752AD2934C58EDEAE338AA?sequence=1). The observed difficulty in P-typing was solved by including a new primer 1T-1GHA—designed based on the 1T-1D degenerate typing primer in our PCR reactions and this additional primer helped genotype the non-typeable strains reported previously in the reference laboratory in Ghana.

Our analysis provided information on how Ghanaian pre-vaccine introduction period G1P[8] and G9P[8] strains relate to a global collection of G1 and G9 strains at the lineage level. The study also resolved the methodological limitations of the standard genotyping techniques employed in the Rotavirus Regional Reference laboratory in Ghana. Genotyping failure attributable to natural variation in primer binding sites could be cited among problems encountered in large surveillance studies. The study highlights the importance of close monitoring of rotavirus genotyping methods as well as regularly updating the primer sequences employed for molecular typing of rotaviruses. The findings also suggest that regions should start to use sequencing data from local rotavirus strains to design and update genotyping primers.

## Supporting information

S1 Table GenBank nucleotide sequence accession numbers of Ghanaian G1P[8]a and G9P[8]a strains. (DOCX)

S2 Table P[8] VP4 nucleotide sequence diversity range based on place and season of sample collection. (DOCX)

S3 Table P[8] VP4 nucleotide sequence diversity range based on G-genotype and season of sample collection. (DOCX)
