# Supplementary material for: Genetic analysis of Ghanaian G1P[8] and G9P[8] rotavirus A strains reveals the impact of P[8] VP4 gene polymorphism on P-genotyping
Source: PLoS One. 2019 Jun 26;14(6):e0218790. doi: 10.1371/journal.pone.0218790 (PMC6594640; doi:10.1371/journal.pone.0218790)
Supplement: S1 Table — (DOCX) [file pone.0218790.s001.docx]

S1 Table: GenBank nucleotide sequence accession numbers of Ghanaian G1P[8]a and G9P[8]a strains

|  | **Sample** | **Year of collection** | **G/P-type** | **Nucleotide sequence accession number** | |
| --- | --- | --- | --- | --- | --- |
|  |  |  |  | **VP4 sequence** | **VP7 sequence** |
| 1 | GHA-00240/DC | 2007 | G1P[8] | KX545213 | ND |
| 2 | GHA-00319/DC | 2008 | G1P[8] | KX545218 | ND |
| 3 | GHA-00328/DC | 2008 | G1P[8] | KX545216 | ND |
| 4 | GHA-00378/DC | 2008 | G1P[8] | KX545215 | ND |
| 5 | GHA-00532/DC | 2008 | G1P[8] | KX545221 | ND |
| 6 | GHA-00324/DC | 2008 | G1P[8] | KX545217 | ND |
| 7 | GHA-00495/DC | 2008 | G1P[8] | KX545220 | ND |
| 8 | GHA-00141/PML | 2008 | G1P[8] | KX545222 | ND |
| 9 | GHA-00329/DC | 2008 | G1P[8] | KX545219 | ND |
| 10 | GHA-5059/EB | 2009 | G1P[8] | KX545246 | LC456072 |
| 11 | GHA-00592/DC | 2009 | G1P[8] | KX545214 | LC456068 |
| 12 | GHA-00850/DC | 2010 | G1P[8] | KX545227 | LC456071 |
| 13 | GHA-0099/M | 2010 | G1P[8] | KX545245 | LC456066 |
| 14 | GHA-0123/P | 2010 | G1P[8] | KX545247 | LC456064 |
| 15 | GHA-0139/P | 2010 | G1P[8] | KX545248 | LC456061 |
| 16 | GHA-0028/K | 2010 | G1P[8] | KX545251 | LC456062 |
| 17 | GHA-0021/K | 2010 | G1P[8] | KX545250 | LC456069 |
| 18 | GHA-00892/DC | 2010 | G1P[8] | KX545229 | LC456065 |
| 19 | GHA-00840/DC | 2010 | G1P[8] | KX545225 | ND |
| 20 | GHA-009I9/DC | 2010 | G1P[8] | KX545231 | LC456063 |
| 21 | GHA-474/AG | 2010 | G1P[8] | KX545224 | LC456060 |
| 22 | GHA-00845/DC | 2010 | G1P[8] | KX545226 | LC456073 |
| 23 | GHA-00702/PML | 2010 | G1P[8] | KX545232 | LC456070 |
| 24 | GHA-00759/PML | 2010 | G1P[8] | KX545235 | LC456077 |
| 25 | GHA-00800/PML | 2010 | G1P[8] | KX545240 | LC456075 |
| 26 | GHA-00789/PML | 2010 | G1P[8] | KX545237 | LC456067 |
| 27 | GHA-00796/PML | 2010 | G1P[8] | KX545239 | LC456074 |
| 28 | GHA-00793/PML | 2010 | G1P[8] | KX545238 | LC456076 |
| 29 | GHA-00713/PML | 2010 | G1P[8] | KX545234 | ND |
| 30 | GHA-0093/M | 2010 | G9P[8] | KX545244 | LC456079 |
| 31 | GHA-00293/LA | 2010 | G9P[8] | KX545223 | LC456085 |
| 32 | GHA-00886/DC | 2010 | G9P[8] | KX545228 | LC456081 |
| 33 | GHA-00894/DC | 2010 | G9P[8] | KX545230 | LC456084 |
| 34 | GHA-00710/PML | 2010 | G9P[8] | KX545233 | LC456078 |
| 35 | GHA-00802/PML | 2010 | G9P[8] | KX545242 | LC456086 |
| 36 | GHA-00801/PML | 2010 | G9P[8] | KX545241 | LC456082 |
| 37 | GHA-00810/PML | 2010 | G9P[8] | KX545243 | LC456083 |
| 38 | GHA-00784/PML | 2010 | G9P[8] | KX545236 | LC456080 |
|  |  |  |  |  |  |

*ND: Not determined*
