# Supplementary material for: Genetic analysis of Ghanaian G1P[8] and G9P[8] rotavirus A strains reveals the impact of P[8] VP4 gene polymorphism on P-genotyping
Source: PLoS One. 2019 Jun 26;14(6):e0218790. doi: 10.1371/journal.pone.0218790 (PMC6594640; doi:10.1371/journal.pone.0218790)
Supplement: S2 Table — (DOCX) [file pone.0218790.s002.docx]

S2 Table: P[8] VP4 nucleotide sequence diversity range based on place and season of sample collection

| **Category based on place and season** | **Sample** | **Year of collection** | **VP4 gene Lineage** | | **P[8] nucleotide sequence diversity variation (%)** |
| --- | --- | --- | --- | --- | --- |
| **DC/2007-2008** | GHA-00240/DC | 2007 | P[8]-Lineage III | | 0.7 – 3.9 |
|  | GHA-00319/DC | 2008 | P[8]-Lineage III | |  |
|  | GHA-00328/DC | 2008 | P[8]-Lineage III | |  |
|  | GHA-00378/DC | 2008 | P[8]-Lineage III | |  |
|  | GHA-00532/DC | 2008 | P[8]-Lineage III | |  |
|  | GHA-00324/DC | 2008 | P[8]-Lineage III | |  |
|  | GHA-00495/DC | 2008 | P[8]-Lineage III | |  |
|  | GHA-00329/DC | 2008 | P[8]-Lineage III | |  |
|  | GHA-00592/DC | 2009 | P[8]-Lineage III | |  |
| **DC/2010** | GHA-00850/DC | 2010 | P[8]-Lineage III | | 0.2 – 3.2 |
|  | GHA-00892/DC | 2010 | P[8]-Lineage III | |  |
|  | GHA-00840/DC | 2010 | P[8]-Lineage III | |  |
|  | GHA-009I9/DC | 2010 | P[8]-Lineage III | |  |
|  | GHA-00845/DC | 2010 | P[8]-Lineage III | |  |
|  | GHA-00886/DC | 2010 | P[8]-Lineage III | |  |
|  | GHA-00894/DC | 2010 | P[8]-Lineage III | |  |
| **PML/2010** | GHA-00702/PML | 2010 | P[8]-Lineage III | | 0.7 – 3.9 |
|  | GHA-00759/PML | 2010 | P[8]-Lineage III | |  |
|  | GHA-00800/PML | 2010 | P[8]-Lineage III | |  |
|  | GHA-00789/PML | 2010 | P[8]-Lineage III | |  |
|  | GHA-00796/PML | 2010 | P[8]-Lineage III | |  |
|  | GHA-00793/PML | 2010 | P[8]-Lineage III | |  |
|  | GHA-00713/PML | 2010 | P[8]-Lineage III | |  |
|  | GHA-00710/PML | 2010 | P[8]-Lineage III | |  |
|  | GHA-00802/PML | 2010 | P[8]-Lineage III | |  |
|  | GHA-00801/PML | 2010 | P[8]-Lineage III | |  |
|  | GHA-00810/PML | 2010 | P[8]-Lineage III | |  |
|  | GHA-00784/PML | 2010 | P[8]-Lineage III | |  |
| **EB/M/P/2009-2010** | GHA-0093/M | 2010 | P[8]-Lineage III | 0 – 2.9 | |
|  | GHA-0176/P | 2010 | P[8]-Lineage III |  |  |
|  | GHA-5059/EB | 2009 | P[8]-Lineage III |  |  |
|  | GHA-0099/M | 2010 | P[8]-Lineage III |  |  |
|  | GHA-0123/P | 2010 | P[8]-Lineage III |  |  |
|  | GHA-0139/P | 2010 | P[8]-Lineage III |  |  |
| **AG/K/2010** | GHA-0028/K | 2010 | P[8]-Lineage III | 0.5 – 3.0 | |
|  | GHA-0021/K | 2010 | P[8]-Lineage III |  |  |
|  | GHA-474/AG | 2010 | P[8]-Lineage III |  |  |
|  |  |  |  |  | |
